# What are the barriers and facilitators to effective health promotion in urgent and emergency care? A systematic review

**DOI:** 10.1186/s12873-022-00651-3

**Published:** 2022-06-03

**Authors:** B. Schofield, U. Rolfe, S. McClean, R. Hoskins, S. Voss, J. Benger

**Affiliations:** 1 University of West of England School of Health and Social Wellbeing, Faculty of Health and Applied Sciences, Glenside Campus, Bristol, BS16 1DD England; 2 Bournemouth University, Faculty of Health and Social Sciences, Bournemouth House, Christchurch Road, Bournemouth, Dorset, BH1 3LH UK; 3 University of the West of England Faculty of Health and Social Sciences, Frenchay Campus, Coldharbour Lane, Bristol, BS16 1QY England

**Keywords:** Paramedic, Emergency medical services, Emergency department, Health promotion, Systematic review

## Abstract

**Background:**

There are potential health gains such as reducing early deaths, years spent in ill-health and costs to society and the health and care system by encouraging NHS staff to use encounters with patients to help individuals significantly reduce their risk of disease. Emergency department staff and paramedics are in a unique position to engage with a wide range of the population and to use these contacts as opportunities to help people improve their health. The aim of this research was to examine barriers and facilitators to effective health promotion by urgent and emergency care staff.

**Methods:**

A systematic search of the literature was performed to review and synthesise published evidence relating to barriers and facilitators to effective health promotion by urgent and emergency care staff. Medical and social science databases were searched for articles published between January 2000 and December 2021 and the reference lists of included articles were hand searched. Two reviewers independently screened the studies and assessed risk of bias. Data was extracted using a bespoke form created for the study.

**Results:**

A total of 19 papers were included in the study. Four themes capture the narratives of the included research papers: 1) should it be part of our job?; 2) staff comfort in broaching the topic; 3) format of health education; 4) competency and training needs. Whilst urgent and emergency care staff view health promotion as part of their job, time restraints and a lack of knowledge and experience are identified as barriers to undertaking health promotion interventions. Staff and patients have different priorities in terms of the health topics they feel should be addressed. Patients reported receiving books and leaflets as well as speaking with a knowledgeable person as their preferred health promotion approach. Staff often stated the need for more training.

**Conclusions:**

Few studies have investigated the barriers to health promotion interventions in urgent and emergency care settings and there is a lack of evidence about the acceptability of health promotion activity. Additional research is needed to determine whether extending the role of paramedics and emergency nurses to include health promotion interventions will be acceptable to staff and patients.

**Supplementary Information:**

The online version contains supplementary material available at 10.1186/s12873-022-00651-3.

## Background

The NHS is committed to using all staff encounters with patients to help individuals significantly reduce their risk of disease [1, 2]. This could reduce early deaths, years spent in ill-health and costs to society and the health and care system. NHS staff have the opportunity to recognise appropriate times and situations in which to engage with patients and help them on the pathway to improving their health and wellbeing. Emergency Department and Emergency Medical Services (Ambulance) staff are in a unique position to engage with a wide range of the population and to use these contacts as opportunities to help people improve their health.

The World Health Organisation describes health promotion as a process of enabling people to increase control over, and to improve, their health [3]. Patient education and effective communication can support individuals to make healthy choices [4]. A range of factors may complicate communication in the Emergency Department (ED). These include variable workloads, crowding, uncertainty and time constraints [5]. Some of these factors also apply to the work environment of paramedics. Whilst the nature of urgent and emergency care may offer challenges when considering health promotion activities, it may also be the ideal environment to create opportunities for a ‘teachable moment’ that will promote subsequent health behaviour change [6]. There is also an economic evidence base for health promotion and disease prevention, as reducing the risk of chronic diseases and injury through interventions aimed at modifying lifestyle risk factors is known to be cost-effective, and could reduce health inequalities [7].

Given the potential health gains, research should be encouraged to organise and deliver effective health promotion interventions in urgent and emergency care settings. The aim of this systematic review was to examine barriers and facilitators to effective health promotion by urgent and emergency care staff. This paper reports on an evidence synthesis relating to the barriers and facilitators to effective health promotion interventions in urgent and emergency care settings. The paper will inform the direction of future research in this field by providing a basis to further explore areas of interest and expressed needs.

## Methods

### Study design

The search methodology and reported findings comply with the relevant sections of the Preferred Reporting Items for Systematic Reviews and Meta-Analyses (PRISMA) statement [8]. Prior to performing this review, a protocol was developed and registered with PROSPERO (registration number CRD42020205180). The research question guiding this systematic review was as follows: “What are the barriers and facilitators to effective health promotion interventions in urgent and emergency care settings?”

Consensus was reached among all reviewers on search syntax, inclusion and exclusion criteria, and the criteria for assessment of validity and relevance in the identified articles.

### Eligibility criteria

Our eligibility criteria followed the Participant, Exposure, Outcome and Study design (PEOS) framework [9]. We only included papers written with publication dates limited from January 2000 to August 2020 in all our information sources. Limiting the search period to 2000 onwards sought to identify all relevant research published in a contemporary timeframe.

### Search strategy

The search strategy was informed by an initial overview of literature in the field and the assistance of a subject librarian. The following bibliographic databases were searched: CINAHL, MEDLINE, Cochrane Central, Cochrane sensitive RCT search strategy, Scopus and PsycINFO on 18th August 2020. The search was repeated on 14th December 2021 to capture any relevant papers published since the original search date. The search included title, abstract, keywords and subject headings to describe the population (paramedics, doctors, nurses and support staff in emergency departments) and the setting (pre-hospital emergency medical (ambulance) services and hospital emergency departments). A detailed strategy for MEDLINE is given in (Table 1) and was adapted to the other databases. All articles that met the search terms were exported from the search engines to the Covidence systematic review management system [10]. Backward chaining within the final sample was reviewed for potentially relevant papers.

**Table 1:** detailed search strategy

MEDLINE, CINAHL, Cochrane Central, Cochrane sensitive RCT strategy, Scopus and PsychInfo databases were searched using the following search terms: 1. promot* or educat* or program* or prevent* or project* or intervent* or strateg* 2. “health education” or “patient education” or “primary prevention” or “health promotion” or “primary health care” or “preventative health care” 3. paramedic* or prehospital or pre-hospital or “emergency care” or “emergency department” or “accident and emergency” or ambulance or “Minor Injury Unit*” or “Urgent Treatment Centre*” 4. S1 N2 S2 N2 S3	

### Selection of studies

A range of research methods was considered including randomised controlled trials, observational studies, surveys and qualitative research. Any literature (quantitative, qualitative or mixed methods) that reported on the facilitators or barriers to health promotion in urgent and emergency care settings was considered for inclusion. This included research papers of any kind but not systematic reviews, literature reviews, editorials, commentaries or letters. Unpublished data was not included.

Based on the inclusion criteria (Table 2), two reviewers (BS and UR) independently screened the titles and abstracts of eligible articles to eliminate articles not meeting the inclusion criteria. Articles not meeting the inclusion criteria based on the title and abstract were excluded at this point. The full text of the agreed included articles was screened independently by the same two reviewers (BS and UR). Articles were excluded at the full-text stage if they did not directly meet the eligibility criteria on closer inspection of the full article. Additionally, references in review articles were screened using the same criteria. Any conflicts during the screening process were resolved through discussion by the two reviewers with reference to the inclusion and exclusion criteria.

**Table 2:** Inclusion criteria

PIEOS categories	Inclusion criteria
Population	1-paramedics 2- emergency department staff 3- patients treated in urgent and emergency care. By urgent and emergency care settings we mean in urgent care settings, a Minor Injury Unit or Urgent Treatment Centre or emergency departments and by Emergency Medical Services (EMS) providers.
Intervention	Any intervention or combination of interventions delivered by urgent and emergency care staff for the promotion of health. The person delivering the intervention and the setting of the intervention was noted.
Exposure	Any health promotion activity.
Outcome	Any barriers or facilitators to undertaking health promotion interventions, including but not limited to engagement with the activity and perceived time constraints, and secondly, how patients and staff view delivery of the interventions in urgent and emergency care settings.
Setting	Pre-hospital setting which is usually the home or normal place of residence of the participant, in a public place or in the ambulance. Emergency Department of a hospital. Minor Injury Unit or similar facility.

Due to heterogeneity between study settings, designs and screening tools used, the included studies have been described narratively [11].

### Data extraction and quality assessment

A bespoke data extraction form was designed in consultation with the review team and piloted on two papers identified during the scoping search. No changes to the data extraction form were recommended following the pilot phase. The data extraction form is reproduced in Additional file 1 Appendix 1. Two authors extracted data separately from the eligible studies (BS and UR). The reviewers conferred and agreed on studies to be included.

The Mixed Methods Appraisal Tool (MMAT) was used to assess the studies for risk of bias, relevance, trustworthiness and results [12]. The authors of the MMAT encourage the provision of a detailed presentation of the ratings of the five criteria within the tool to reflect the quality of the included studies. In this review the studies were ranked as high (all criteria met), medium (four out of five criteria met) and low (three or less criteria met). Much of the MMAT assessment process focuses on the risk of bias in the study under consideration, and therefore studies judged as low quality are at the highest risk of bias when this tool is applied. Conflicts in risk of bias assessment were resolved through discussion by the two reviewers and with reference to the appraisal tool. The methodological quality of each study was independently analysed by two authors (BS and UR). No studies were excluded based on quality assessment. The quality rankings for each study are presented in Table 3.

**Table 3:** Characteristics of included studies (*n =* 19)

1st author (Year) Country [ref]	Design	Methods/ Data sources	Sample	Barriers/ Facilitators/ Preferences/ Outcomes	Assessment of quality (MMAT) [high/medium/low]
Gielen A.C. (2020) US [13]	Randomised, controlled trial	Participants were assigned randomly to receive either a personalised and stage-tailored safety report (intervention group) or a personalised but otherwise generic report on other child health topics (control group). Follow-up interviews were conducted by telephone 2 to 4 weeks and 4 months after enrolment by interviewers	1412 parents with children who were age-eligible according to the triage sheet were approached; 239 (17%) were ineligible, 201 (14%) refused to participate, and 69 (5%) were missed by the recruiters. 901 parents were enrolled (448 in the intervention group and 453 in the control group). Follow-up rates were 86% for the intervention group (*n =* 384) and 83% for the control group (*n =* 375). Total (*n =* 759)	This technology is feasible for use in a busy ED with minimal intrusion into patient flow; significant improvements in safety knowledge resulted from the intervention.	low
Koonce T.Y. (2011) US [14]	Randomised trial	Standard care discharge instructions or standard care combined with information individualised to their learning-style preference. 2 weeks post-visit knowledge survey.	ED patients aged eighteen or older, able to speak and read English, and able to provide telephone contact information were eligible for the study A total of 185 patients were initially identified inclusion. Of these, 109 patients were excluded for not meeting all inclusion criteria, refusal to participate, and other reasons. 76 patients were randomized to either the control or intervention groups. Seven patients in each arm were unable to be reached for follow up. (*n =* 76)	Learning style–tailored information patients perceived that the materials increased their understanding; demonstrated the feasibility of implementing a learning-style approach to patient education in the ED. Provides a framework for developing customised information prescriptions that can be broadly adapted for use across various health care conditions.	medium
Smith S. (2008) Australia [15]	Prospective randomised controlled trial	two inner-city Australian teaching hospital EDs. Patients received either standard patient education or patient-centred education (PCE). Both groups received a six-topic curriculum. PCE patients reordered the topics according to their own priority controlling the order of education.	Adult patients presenting to two EDs with acute asthma during a 12 month period – 148, two refused participation, (*n =* 146)	Trend of better asthma control for the PCE group with fewer ED visits within 4 months of being educated; PCE provides potential for patients to be active participants; brief, patient-centred education processes using a basic chronic disease guideline curriculum may be of value for people who are treated, educated and discharged from the ED	low
Chan Y-F. (2006) US [16]	Randomized before-after pre-test/post-test trial with viewing of a stroke video serving as the intervention. Follow-up telephone interview using the same questionnaire for both cohorts	Subjects were randomized into two arms: those watching a 12-min educational video on stroke (video group) and those not undergoing an intervention (control group). Both groups were administered a 13-question quiz covering different stroke-related issues, but only the video group received this same test again after completion of the educational program. Those enrolled were contacted after 1 month to determine knowledge retention via the same test.	A convenience sample of research subjects was recruited from ED waiting areas (*n =* 198)	Even at the 1-month follow-up, the video group had significantly higher test scores than the control group. Educational video may be a valuable and relatively low-cost tool for focused patient education in the ED.	low
Robson S. (2020) UK [17]	Multicentre, structured survey	Staff who verbally consented received a paper questionnaire.	All doctors and nursing staff at two teaching and two district general hospitals (*n =* 423)	Staff felt health promotion was important in the ED; one third of staff felt their role involved providing brief interventions and to screen patients for modifiable risk factors and identify those suitable for interventions; leaflets were the most popular choice; staff believed that health promotion interventions could be delivered through a variety of methods and modes of delivery; drug and alcohol misuse were the most appropriate risk factors to discuss in ED, especially if related to ED presentation; interventions in the ED were more appropriate when risk factors were directly related to ED presentation	medium
Bernstein J. (2017) US [18]	Survey then brief intervention delivered, followed by appropriate referral	Patients 14–21 yrs. screened for high-risk behaviour (survey), received a brief intervention with written handout and a list of community support programmes	2149 patients screened, 834 screened positive for at least 1 health risk and received a referral, 636 received a brief intervention and 546 referred for specialist treatment	Convincing staff that prevention-based services in the ED could be helpful; educating staff; use of a Health Promotion Advocate integrated with the ED team	low
Coombs N.M. (2016) Australia [19]	Quantitative, pre and post-test questionnaire comparison study	Data were collected before and after the implementation of a staff education session, including introducing a new education tool; ED-HOME.	Convenience sample of 14 ED nurses - (102 permanent nursing staff)	Using the structured tool led to improvement in confidence in providing education; more structured personalised education being given. If emergency nurses feel more confident with their educating practices and by using a structured format, patients may benefit from better quality patient education.	low
Mieschke H. (2014) US [20]	Survey	EMT-delivered patient education intervention for community residents who called 911 for a non-life- threatening event on blood pressure management, blood glucose management or fall prevention	Firefighter emergency medical technicians (*n =* 822)	EMS providers reported they were most likely to hand out the pamphlet to patients in private residences who were treated and left at the scene; less likely if language barrier, in care centre or nursing home.	medium
Lynagh M. (2010) Australia [21]	Self-administered questionnaire	A cross-sectional, descriptive survey	A stratified random sample of 500 ambulance officers from all four sectors across New South Wales, Australia, were selected and invited to participate in the study. 264 officers (out of 500) participated in the study, providing a response rate of 53%. (*n =* 264)	Need to receive training on how to deal with alcohol-affected patients, how to make referrals and to provide brief advice; not enough time to discuss issues; patient might get angry; half believed their role included health promotion Ambulance officers are ideally situated to identify and detect ‘at-risk’ drinkers because of the apparent high prevalence of alcohol-related call-outs, and are willing to screen for problem drinking.	medium
Delgado K.M. (2010) US [22]	Survey	Four EDs surveyed interest in 28 health conditions and topics	1321 eligible subjects – consecutive adult patients and visitors presenting to ED. 1010 (76%) completed the survey, of whom 56% were patients and 44% were visitors	Most interested in health education on stress, depression, exercise, and nutrition; preferred the traditional form of books and brochure. Learning preferences of ED population should be incorporated into future plans.	low
Walton M. (2008) US [23]	Self-administered survey and follow up interview one month post ED visit	Adolescents were surveyed and referred to a violence prevention website. Website login data were tracked by specific logon ID one month post-ED visit.	Adolescents (ages 12–17) visiting ED (*n =* 115)	Twice as many participants stated they logged on as did; the Internet may provide a unique solution to busy clinicians providing health interventions.	low
Cross R. (2005) UK [24]	Q methodology	A within-subjects design using Q methodology	Nurses working in the ED (*n =* 11)	Positive view of health promotion and the ED nurses’ role; lack of support from management; lack of knowledge and skills; ED is a suitable environment for health promotion. It is not possible to generalize the findings of this study due to the small number of participants.	low
Rhodes K.V. (2001) US [25]	Self-administered computer survey	Controlled trial, with alternating assignment of patients to a computer intervention (prevention group) or usual care	542 ED adult patients with non-urgent conditions were eligible, 89% participated (*n =* 470)	ED patients were very accepting of this technology and interested in using their waiting time as an opportunity to receive health information; patients receiving the computer intervention were more likely than the control group to remember being given health advice 1 week after the ED visit. Computer methodology may enable staff to use patient waiting time for health promotion and to target at-risk patients for specific interventions.	low
Williams J.M. (2000) US [26]	Survey	Two questionnaires posted 4 weeks apart and the responses to these	Survey sent to all 165 members of the West Virginia Chapter of the American College Of Emergency Physicians (*n =* 56)	Physicians identified as being responsible for health education but felt ill prepared; pessimistic about success in helping patients change behaviours; smoking most commonly discussed	medium
Hawkins E.R. (2007) US [27]	Retrospective review of injury prevention surveys	Paramedics were trained to use the injury prevention survey during home visits; homes with newborn infants identified and contacted; home visits agreed; survey served as a tool for home visit	Paramedic home visits with reports (*n =* 262)	Paramedics can recognize common hazards in the home and provide education and mitigation to reduce risks of paediatric injury; paramedics can distribute home safety devices in a community injury prevention program	low
Sheahan S.L. (2000) US [28]	Retrospective review of medical records; two-group comparative study – nurse practitioners and doctors	Researchers examined random-stratified medical records of 305 non-acute ambulatory patients for selected health risk factors, including smoking, alcohol use, elevated blood pressure, obesity, and dental caries.	Emergency service medical records of a random-stratified sample of nonacute ambulatory adult patients for selected health risk factors (*n =* 305)	Records showed a lack of documentation of assessments of weight and tobacco and alcohol use; only 22% of adults with non-acute health problems received appropriate health promotion counselling; doctors documented more health risks than nurse practitioners	low
Martin A. (2016) Canada [29]	Observational ethnographic approach with qualitative interviews	Qualitative data through informal discussions, semi-structured interviews and direct observation of interactions between consumers and community paramedics.	Purposive sampling of adult community members (patients, relatives and carers) (*n =* 14)	Acceptance of paramedics in non-traditional preventative health care roles.	low
Shoqirat N. (2013) Jordan [30]	Qualitative semi-structured interviews	Interview transcripts	Convenience sample of 15 nurses in a Jordanian emergency department	Not our role ‘let other people do it’; nurses’ lack of competency in health promotion; fear of violence; lack of a policy and protocols; patients’ beliefs . Cultural issues and challenges may be a barrier in expanding the role of health promotion in EDs.	low
Bensberg M. (2003) Australia [31]	Focus groups with ED staff and a workshop for health professionals who were external to EDs	Seven focus groups were held, one at each of the participating EDs; one workshop representing 5 EDs	Focus groups (*n =* 76) Workshop (*n =* 55)	Patients may not be willing to lengthen their stay at the ED to partake in health promotion activities; should be occurring further ‘upstream’; ethics of behaviour change and perceived coercion; cost; lack of staff understanding	medium

## Results

### Study selection

Overall, research into barriers and facilitators of health promotion activity in urgent and emergency care settings was found to be limited. No relevant research was identified regarding paramedics. It was therefore necessary to increase the scope of the review to include community paramedicine programmes in rural settings in North America and Australia. Whilst these programmes are not directly transferable to the role of paramedics more generally, they are able to demonstrate the acceptability of this non-traditional role, which includes health promotion, amongst the wider paramedic profession.

154 papers were identified through database searching. Following the removal of duplicates, 108 records were reviewed by title and abstract. Of these, 63 were removed. 45 records were assessed for eligibility based on a full text review. 26 were excluded, with 19 records being included in the review. Inter-rater agreement for full text exclusion was strong (k = 0.86). A flow-chart of the search strategy and selection is presented in Additional file 1 Appendix 2.

Studies took place in the following countries: 11 in the US [13, 14, 16, 18, 20, 22, 23, 25–28], 1 in Jordan [30], 2 in the UK [17, 24], 4 in Australia [15, 19, 21, 31] and 1 in Canada [29]. The characteristics of the included studies and participants are described in Table 3.

### Data synthesis

The 19 studies were published between 2000 and 2020 and included a range of populations and research methodologies. Ten studies were surveys, four were randomised controlled trials, two were retrospective reviews of records and three were qualitative interviews/focus groups. Sample sizes ranged from 2149 to 11 participants. Four themes capture the narratives of the included research papers: 1) should it be part of our job?; 2) risk of offending patients; 3) format of health education; 4) competency and training needs. These four themes capture the reported barriers and facilitators to effective health promotion interventions in urgent and emergency care settings.

#### Should it be part of our job?

In general staff support health promotion taking place in the ED. [17, 18, 21, 24, 26, 28] Paramedics in rural communities and emergency services technician firefighters also see health promotion as an acceptable part of their jobs [20, 27, 29]. However, ED nurses in one Jordanian study felt it was not part of their role [30].

Whilst nurses felt that health promotion was part of their role, they reported providing health promoting advice less than half the time when these interactions would have been indicated. They reported lack of time and a lack of support systems for patient follow up as barriers [18]. Although ED doctors reported feeling responsible for promoting the health of their patients, only a minority reported routinely screening and counselling their patients with identified modifiable risk factors. Most reported not feeling confident in their ability to help patients change their behaviour [26]. In one study doctors reportedly offered health promotion intervention more often than nurses. Time constraints and a lack of health promotion infrastructure in the ED were cited as challenges to intervention delivery [17]. Patients and carers attended to by community paramedics accepted paramedics in a non-traditional preventative healthcare role [29].

#### Staff comfort in broaching the topic

The health conditions of interest to ED patients in one study were stress and depression and among the health topics, participants were most interested in exercise and nutrition [22]. Smoking is the health topic most commonly discussed according to ED doctors in one study [26]. Whilst ED staff in another study stated that drug and alcohol misuse were the most appropriate risk factors to discuss in ED and that the interventions in the ED were most appropriate when risk factors were directly related to the ED presentation [17]. Paramedics had success with injury prevention advice as part of their role in community paramedicine [27]. The recording of health risks and counselling was noted in only 22% of nonacute patients with one or more modifiable risk factors; with doctors documenting more health risks than nurses [28].

Whilst 20% of all calls for an ambulance service involve alcohol, not many ambulance officers ask the patients they attend about quantity and frequency of alcohol use [21].

#### Format of health education

Educational, and to a lesser extent behavioural change, approaches are the main forms of health promotion described in the urgent and emergency care setting [32]. Patients and visitors stated they preferred traditional forms of books and leaflets to support the information they were given on health-related topics [22]. An educational video used during ED waiting was shown to improve knowledge and act as an acceptable low-cost teaching tool for focused patient education that may allow clinicians to use patient waiting time for health promotion [16, 25]. The use of learning style-tailored information led to patients perceiving improved knowledge [14]. Using a structured education tool improved nurse confidence in undertaking personalised education prior to discharge from the ED. [19] A computer kiosk to promote child safety in a randomised controlled trial in an urban paediatric emergency department demonstrated the applicability of computer technology for education in a busy ED. [13]

Inadequate patient education has been cited as a potential cause of re-attendance of asthma patients to the ED. A randomised study aimed to compare the effectiveness of patient-centred education (PCE) and standard asthma patient education on ED re-attendance. PCE patients had fewer re-attendances at 4 and 12 months. A learner-centred approach to education may be useful in reducing re-attendances to the emergency department [15]. Internet referrals may provide a potential solution to limited staff time in emergency departments for health education [23].

#### Competency and training needs

There was a statement of continued need for education in health promotion roles in those studies where staff views were collected [19, 21, 24, 26, 30, 31]. Nurses felt they lacked competency [30], were less knowledgeable on some health topics than others [24, 26, 31], and requested a structured approach [16]. Paramedics requested specific training to deal with patients affected by excessive alcohol intake [21]. Staff were concerned that existing health promotion interventions were not systematic and had not been evaluated and risked becoming a marginalised part of their work [31]. Lack of health promotion knowledge, lack of time and not wanting to extend a patient’s stay in the ED were reported as barriers.

## Discussion

Nineteen studies with varying designs were identified as relevant for our exploration of barriers and facilitators to effective health promotion in urgent and emergency care. The evidence base is not well developed. There is limited evidence describing the barriers to health promotion activities in EDs, and facilitators are particularly poorly captured. Two literature reviews suggest that educational interventions in the ED are both possible and feasible, while indicating that additional research is needed to provide a more substantial evidence base from which to identify effective approaches designed specifically for this healthcare setting [33, 34]. This review supports these statements and highlights a need for further research in this area, in particular to understand the views of staff and patients on the potential for an expansion of the role of ED nurses and paramedics.

Almost all relevant research has suggested that urgent and emergency care staff view health promotion as a part of their job, however time restraints and a lack of knowledge and experience are identified as barriers to undertaking health promotion interventions. If emergency nurses feel more confident in their educating practices, and are supported by a structured format, patients may benefit from better quality patient education provided in the ED. The provision of a health promotion infrastructure in the ED will be a positive step towards providing a standard approach and is likely to include training and support pathways for ED staff to ensure that health promotion is an integral part of their role.

Whilst patients have reported that the health promotion topics they are most interested in are exercise and nutrition, ED staff shy away from health promotion interventions relating to weight management, diet and exercise [18, 22, 26] There may be worries around seeming insensitive to patients and further stigmatising patients that prevent staff from engaging in these interactions. Staff in general report providing health promotion interventions on blood pressure management, smoking and alcohol use. ED staff agree that health promotion interventions are most effective if related to an acute ED presentation. This may be one reason why diet and weight management are not seen as appropriate interventions in this setting. A study of General Practitioners and practice nurses in the UK on talking to primary care patients about weight found that staff had concerns about raising the issue of overweight; the most common being that patients would react emotionally to the message [35].

Patients reported receiving books and leaflets as well as speaking with a knowledgeable person as their preferred health promotion approach. A systematic review of the effectiveness of traditional media (leaflet and poster) to promote health in a community setting, demonstrated that traditional health promotion media such as leaflets and posters are still useful in the current digital era, especially for adult respondents [36].

A number of studies have demonstrated the feasibility of video and internet use in the ED waiting areas as acceptable methods of patient education. A disease-specific educational video may be a relatively low-cost tool for focused patient education in the ED waiting room. These combined approaches may have the potential to offer improved outcomes for patients visiting the ED but adopting them will require structural and cultural changes. A systematic review of the effectiveness of video-based education in modifying health behaviours demonstrated that for certain health messages and conditions video interventions appear to be effective [37].

Patient discharge from the ED appears to be an effective time to maximise engagement with ED recommendations and improve self-care according to the literature reviewed. A variety of potential teaching methods and teaching materials have been used in the ED; however, it is still unclear which of these are most effective, and for which subgroup of the population [38]. Given the potential for health gains, research should examine how to organise and deliver the most effective patient education in the ED.

The role of the paramedic in health promotion is beginning to receive some attention [39, 40]. Health promotion and healthy lifestyle interventions are outlined in the Paramedic Specialist in Primary and Urgent Care Core Capabilities Framework produced by the College of Paramedics [41]. The included literature demonstrates support from community paramedics and emergency medical technicians in Canada, US and Australia for the expanded role of health promotion as part of their activities when treating patients in the community [20, 21, 27, 29]. This literature highlights how paramedics in the ambulance service may be able to adapt to health promotion activities when treating and discharging patients at home.

The themes identified in this review can be both facilitators and barriers to undertaking effective health promotion interventions in urgent and emergency care settings. If staff view health promotion as part of their role it will be a facilitator to undertaking effective health promotion interventions in urgent and emergency care settings. Conversely, if staff feel there is a tension between their role as urgent and emergency care practitioners and health promotion, it is likely to act as a barrier with restraints on time and lack of confidence having an impact on the likelihood of staff engagement with health promotion interventions in these settings. On the theme of staff comfort of broaching the topic, if staff view the health promotion discussion as sensitive, it will act as a barrier, and they are less likely to engage in the conversation. Conversely, if staff feel comfortable with the health promotion topic it will act as a facilitator, and they are likely to engage with the patient more readily. Additionally, if the format of the health education approach is patient-centred, and appropriate for their learning needs, it is likely to act as a facilitator to undertaking effective health promotion interventions in urgent and emergency care settings. Conversely, inappropriate health education approaches could act as a barrier in these settings. Finally, if staff feel they lack competency and training in health promotion it is likely to act as a barrier to undertaking effective health promotion interventions in urgent and emergency care settings. Conversely, staff who feel they have adequate competency and training will be more likely to undertake effective health promotion interventions.

Heterogeneity in study settings, designs and the screening tools used in the included studies affects the conclusions and recommendations of this systematic review as it decreases the generalisability of the findings to the management of health promotion interventions in the urgent and emergency care settings [42, 43]. This variability in participants and methodological diversity is the reason we decided to describe the included studies narratively, rather than attempting any form of statistical analysis.

The lack of evidence on the acceptability of health promotion for patients and service providers in urgent and emergency care settings, coupled with an imperative to ensure staff talk to the public they are treating about their health and wellbeing across all health and social care organisations, requires further exploration. There is a need to efficiently integrate existing information and determine the extent to which findings are generalisable across health care settings. This will guide future research on health promotion in urgent and emergency care to generate evidence on patient benefit. This review draws together a disparate literature to identify themes and create an overview with pointers towards future research that has the potential to change practice.

### Limitations

This review was limited to research papers published since January 2000. There is a risk of missing grey literature and relevant literature published prior to 2000. The wide range of methods, countries and interventions described in the included studies makes generalisation difficult.

### Future directions

Future research is necessary to define and understand the barriers and facilitators to health promotion interventions in urgent and emergency care settings. Current evidence does not support changes to clinical practice, and further research is required to build an evidence base that will justify the introduction of new interventions and staff behaviours when caring for patients in emergency care. We anticipate existing clinical practice will be modified if high quality research demonstrating the clinical and cost effectiveness of one or more defined interventions relevant to a particular health system is published.

## Conclusions

Few studies have investigated the barriers to health promotion interventions in urgent and emergency care settings. The papers reviewed in this article demonstrate a willingness amongst staff in urgent and emergency care to undertake health promotion activities. The studies included highlight what emergency department nurses may need to undertake the role of health promotion in their clinical setting. The included papers are mainly from the US, Canada and Australia and there are cultural considerations that need to be considered in future research. Additional research is needed to determine whether extending the role of paramedics and emergency nurses to include health promotion interventions will be acceptable to staff and patients, and to generate an emerging evidence base that will direct future research and practice.

## Supplementary Information

**Additional file 1.**

## Data Availability

The datasets used and/or analysed during the current study are available from the corresponding author on reasonable request.

## References

[1] - NHS The Long-Term Plan January 2019 (Accessed 3 March, 2021).

[2] - Making Every Contact Count (MECC): Consensus statement. Produced by Public Health England, NHS England and Health Education England, with the support of partner organisations identified below. April 2016 (Accessed 3 March, 2021).

[3] - https://www.who.int/health-topics/health-promotion (Accessed 2 March, 2021).

[4] Totnes K, Tilford S (1994). Health education: effectiveness, efficiency and equity.

[5] Eisenberg EM, Murphy AG, Sutcliffe K (2005). Communication in emergency medicine: implications for patient safety. Commun Monogr.

[6] Flocke SA, Clark E, Antognoli E, Mason MJ, Lawson PJ, Smith S (2015). Cohen DJ teachable moments for health behaviour change and intermediate patient outcomes.

[7] Merkur S, Sassi F, McDaid D. Promoting health, preventing disease: is there an economic case? (2013): WHO. https://www.euro.who.int/__data/assets/pdf_file/0004/235966/e96956.pdf

[8] Moher D, Liberati A, Tetzlaff J (2009). Preferred reporting items for systematic reviews and meta-analyses: the PRISMA statement. J Clin Epidemiol.

[9] Munn Z, Stern C, Aromataris E, Lockwood C, Jordan Z (2018). What kind of systematic review should I conduct? A proposed typology and guidance for systematic reviewers in the medical and health sciences. BMC Med Res Methodol.

[10] - https://www.covidence.org/

[11] Popay J, Roberts H, Sowden A, Petticrew M, Arai L, Rodgers M, et al. Guidance on the conduct of narrative synthesis in systematic reviews. ESRC Methods Programme. 2006.

[12] Hong QN, Pluye P, Fàbregues S, Bartlett G, Boardman F, Cargo M, Dagenais P, Gagnon M-P, Griffiths F, Nicolau B, O’Cathain A, RousseauM C, Vedel I (2018). Mixed Methods Appraisal Tool (MMAT), version. Registration of copyright (#1148552).

[13] Gielen A, McKenzie L, McDonald E, Shields W, Wang M-C, Cheng Y-J, et al. Using a Computer Kiosk to Promote Child Safety: Results of a Randomized, Controlled Trial in an Urban Pediatric Emergency Department. PEDIATRICS. 2007;120(2). 10.1542/peds.2006-2703 17671059

[14] Koonce TG, N. Storrow A. A pilot study to evaluate learning style–tailored information prescriptions for hypertensive emergency department patients. J Med Libr Assoc. 2011;99(4). 10.3163/1536-5050.99.4.005 PMC3193368 22022222

[15] Smith S, Mitchell C, Bowler S (2008). Standard versus patient-centred asthma education in the emergency department: a randomised study. Eur Respir J.

[16] - Chan Y-F. Lavery R. Fox F. Kwon R. Zinzuwadia S. Massone R. Livingston D. Effect of an educational video on emergency department patient stroke knowledge. J Emerg Med, 34, 2, 215–220, 2008. 10.1016/j.jemermed.2007.04.003 17976815

[17] Robson S, Stephenson A, McCarthy C, Lowe D, Conlen B, Gray A (2020). Identifying opportunities for health promotion and intervention in the ED. Emerg Med J.

[18] Bernstein J, Dorfman D, Lunstead J, Topp D, Mamata H, Jaffer S, Bernstein E (2017). Reaching adolescents for prevention: the role of pediatric emergency department health promotion advocates. Pediatric Emergency Care Issue.

[19] Coombs N, Porter J, Beauchamp A (2016). ED-HOME: Improving educator confidence and patient education in the Emergency Department. Australas Emerg Nurs J.

[20] Meischke H, Stubbs B, Fahrenbruch C, Phelan E (2014). Factors associated with the adoption of a patient education intervention among first responders, King County, Washington, 2010–2011. Prev Chronic Dis.

[21] Lynagh M, Sanson-Fisher R, Shakeshaft A (2010). Alcohol-related harm: perceptions of ambulance officers and health promotion actions they do and would do. Health Promot J Aust.

[22] Delgado K, Ginde A, Pallin D, CamargoC J. Multi-center Study of Preferences for Health Education in the Emergency Department Population. Acad Emerg Med. 2010;17(6). 10.1111/j.1553-2712.2010.00764.x 20624148

[23] Walton M, Cunningham R, Xue Y, Trowbridge M, Zimmerman M, Maio R (2008). Internet Referrals for Adolescent Violence Prevention: An Innovative Mechanism for Inner-city Emergency Departments. J Adolesc Health.

[24] Cross R (2005). Accident and emergency nurses’ attitudes towards health promotion. J Adv Nurs.

[25] Rhodes K, Lauderdale D, Stocking C, Howes D, Roizen M, Levinson W. Better Health While You Wait: A Controlled Trial of a Computer-Based Intervention for Screening and Health Promotion in the Emergency Department. Ann Emerg Med. 2001;37(3). 10.1067/mem.2001.110818 11223765

[26] Williams J, Chinnis A, Gutman D. Health promotion practices of emergency physicians. Am J Emerg Med. 2000;18(1). 10.1016/s0735-6757(00)90041-x 10674525

[27] Hawkins E, Brice J, Overby B. Findings from an emergency medical services pediatric injury prevention program. Pediatr Emerg Care. 2007;23. 10.1097/PEC.0b013e318159ffd9 18007209

[28] Sheahan S (2000). Documentation of health risks and health promotion counselling by emergency department nurse practitioners and physicians. J Nurs Scholarsh.

[29] Martin A, O’Meara P, Farmer J (2016). Consumer perspectives of a community paramedicine program in rural Ontario. Aust J Rural Health.

[30] Shoqirat N. ‘Let other people do it...’: the role of emergency department nurses in health promotion. J Clin Nurs. 23:232–42. 10.1111/jocn.12383. 10.1111/jocn.12383 24313938

[31] Bensberg M, Kennedy M, Bennetts S (2003). Identifying the opportunities for health promoting emergency departments. Accid Emerg Nurs.

[32] Ewles L, Simnett I (2003). Promoting health; a practical guide.

[33] Wei H, Camargo C. Patient Education in the Emergency Department. Acad Emerg Med. 2000;7(6). 10.1111/j.1553-2712.2000.tb02052.x 10905653

[34] Szpiro K, Harrison M, Van Den Kerkhof E, Lougheed D (2008). Patient Education in the Emergency Department: A Systematic Review of Interventions and Outcomes. Adv Emerg Nurs J Issue.

[35] Michie S (2007). Talking to primary care patients about weight: A study of GPs and practice nurses in the UK. Psychol Health Med.

[36] Barik AL, Purwaningtyas RA, Astuti D (2019). The effectiveness of traditional media (leaflet and poster) to promote health in a community setting in the digital era: A systematic review. Jurnal Ners.

[37] Tuong W, Larsen ER, Armstrong AW (2014). Videos to influence: a systematic review of effectiveness of video-based education in modifying health behaviors. J Behav Med.

[38] Pétré B, Marga A, Servotte C, Guillaume M, Gagnayre R, Ghuysen A (2020). Patient education in the emergency department: take advantage of the teachable moment. Adv Health Sci Educ.

[39] Schofield B, McClean S. Paramedics and health promotion. Perspect Public Health. 2021. 10.1177/17579139211053363. 10.1177/17579139211053363 PMC9047089 34674589

[40] - Association of Ambulance Chief Executives. Working together with ambulance services to improve public health and wellbeing, 2017. Available online at: https://tinyurl.com/ql82kjm (last accessed 17 March 2021).

[41] - https://skillsforhealth.org.uk/info-hub/paramedic-specialist-in-primary-and-urgent-care-2019/ (Accessed 10 March, 2021).

[42] Gagnier JJ, Morgenstern H, Altman DG (2013). Consensus-based recommendations for investigating clinical heterogeneity in systematic reviews. BMC Med Res Methodol.

[43] Imrey PB (2020). Limitations of Meta-analyses of Studies With High Heterogeneity. JAMA Netw Open.

