# Supplementary material for: What are the barriers and facilitators to effective health promotion in urgent and emergency care? A systematic review
Source: BMC Emerg Med. 2022 Jun 3;22:95. doi: 10.1186/s12873-022-00651-3 (PMC9164411; doi:10.1186/s12873-022-00651-3)
Supplement: Supplementary file 1 — Additional file 1. [file 12873_2022_651_MOESM1_ESM.docx]

**Appendix 1 - data extraction form**

Reviewer: Date:

study citation:

author/year:

country of study:

publication type:

study aim:

study population characteristics:

study design:

setting:

inclusion criteria:

exclusion criteria:

sample size:

sampling technique:

data source:

analysis:

key barriers identified to health promotion activities in these settings:

key facilitators identified to health promotion activities in these settings:

patient attitudes to health promotion in these settings:

staff attitudes to health promotion in these settings:

1. Is there congruity between the stated philosophical perspective and the research methodology?

Yes □ No □ Unclear □ Not applicable □

1. Is there congruity between the research methodology and the research question or objectives?

Yes □ No □ Unclear □ Not applicable □

1. Is there congruity between the research methodology and the methods used to collect data?

Yes □ No □ Unclear □ Not applicable □

1. Is there congruity between the research methodology and the representation and analysis of data?

Yes □ No □ Unclear □ Not applicable □

1. Is there congruity between the research methodology and the interpretation of results?

Yes □ No □ Unclear □ Not applicable □

1. Is there a statement locating the researcher culturally or theoretically?

Yes □ No □ Unclear □ Not applicable □

1. Is the influence of the researcher on the research, and vice- versa, addressed?

Yes □ No □ Unclear □ Not applicable □

1. Are participants, and their voices, adequately represented?

Yes □ No □ Unclear □ Not applicable □

1. Is the research ethical according to current criteria or, for recent studies, and is there evidence of ethical approval by an appropriate body?

Yes □ No □ Unclear □ Not applicable □

1. Do the conclusions drawn in the research report flow from the analysis, or interpretation, of the data?

Yes □ No □ Unclear □ Not applicable □

**Overall appraisal:**

**Include □ Exclude □ Seek further info □**

Comments (Including reason for exclusion):

**Appendix 2 - study selection flow-chart**

Records identified through database searching
(n = 154)

Full-text articles excluded, with reasons
(n = 26)

8 Wrong intervention

3 Wrong setting

3 can't access article

3 literature reviews

3 outside of date parameter

2 book not a journal article

2 reflections not research

2 thesis not a journal article

Records excluded
(n = 63)

Records screened
(n = 108)

Records after duplicates removed
(n = 108)

Studies included in analysis
(n = 19)

Full-text articles assessed for eligibility
(n = 45)

## Included

## Eligibility

## Screening

## Identification
